# Detection and Genomic Characterization of a *Morganella morganii* Isolate From China That Produces NDM-5

**DOI:** 10.3389/fmicb.2019.01156

**Published:** 2019-05-28

**Authors:** Xiaobing Guo, Yuting Rao, Lihua Guo, Hao Xu, Tao Lv, Xiao Yu, Yunbo Chen, Na Liu, Huiming Han, Beiwen Zheng

**Affiliations:** ^1^Department of Laboratory Medicine, The First Affiliated Hospital of Zhengzhou University, Zhengzhou, China; ^2^State Key Laboratory for Diagnosis and Treatment of Infectious Diseases, Collaborative Innovation Center for Diagnosis and Treatment of Infectious Diseases, The First Affiliated Hospital, College of Medicine, Zhejiang University, Hangzhou, China; ^3^Basic Medical College, Beihua University, Jilin City, China; ^4^The Clinical Immunology Research Center, Beihua University, Jilin City, China

**Keywords:** *bla*_NDM–5_, IncX3, *Morganella morganii*, complete genome sequence, comparative genomic analysis

## Abstract

The increasing prevalence and transmission of the carbapenem resistance gene *bla*_NDM–5_ has led to a severe threat to public health. So far, *bla*_NDM–5_ has been widely detected in various species of *Enterobacterales* and different hosts across various cities. However, there is no report on the *bla*_NDM–5–_ harboring *Morganella morganii.* In January 2016, the first NDM-5-producing *Morganella morganii* L241 was found in a stool sample of a patient diagnosed as recurrence of liver cancer in China. Identification of the species was performed using 16S rRNA gene sequencing. Carbapenemase genes were identified through both PCR and sequencing. To investigate the characteristics and complete genome sequence of the *bla*_NDM–5_-harboring clinical isolate, antimicrobial susceptibility testing, S1 nuclease pulsed field gel electrophoresis, Southern blotting, transconjugation experiment, complete genome sequencing, and comparative genomic analysis were performed. *M. morganii* L241 was found to be resistant to broad-spectrum cephalosporins and carbapenems. The complete genome of L241 is made up from both a 3,850,444 bp circular chromosome and a 46,161 bp self-transmissible IncX3 plasmid encoding *bla*_NDM–5_, which shared a conserved genetic context of *bla*_NDM–5_ (ΔIS*3000*-ΔIS*Aba125*-IS*5*-*bla*_NDM–5_-*ble*-*trpF*-*dsbC*-IS*26*). BLASTn analysis showed that IncX3 plasmids harboring *bla*_NDM_ genes have been found in 15 species among *Enterobacterales* from 13 different countries around the world thus far. In addition, comparative genomic analysis showed that *M. morganii* L241 exhibits a close relationship to *M. morganii subsp. morganii* KT with 107 SNPs. Our research demonstrated that IncX3 is a key element in the worldwide dissemination of *bla*_NDM_-_5_ among various species. Further research will be necessary to control and prevent the spread of such plasmids.

## Introduction

*Morganella morganii* is a facultative anaerobic Gram-negative bacterium, the representative strain of the genus *Morganella* (Liu et al., 2016). This bacterium tends to colonize in the intestinal tracts of humans, mammals and reptiles as part of the normal flora, and is often found in the environment (Lee et al., 2009). It is noteworthy that *M. morganii* is an opportunistic pathogen, but the disease spectrum associated its infections is broad, mainly including sepsis (Seija et al., 2015), abscess (Zaid et al., 2013), urinary tract infection (Jamal et al., 2015), and bacteremia (Ghosh et al., 2009). Furthermore, *M. morganii* can harbor ESBLs and carbapenemase, which adds resistance to multiple antibiotics and has resulted in a high mortality rate in some infections (Liu et al., 2016). There are existing reports of the detection of New Delhi metallo-β-lactamase-1 (NDM-1) (Olaitan et al., 2014), *Klebsiella pneumonia* carbapenemases-2 (KPC-2) (Shi et al., 2011), and Metallo-β-lactamase VIM-1 (Tsakris et al., 2007) in *M. morganii*. However, to date, NDM-5-producing *M. morganii* has not been described.

NDM-5 was first identified in *Escherichia coli* from a patient who had been hospitalized in India in 2011 (Hornsey et al., 2011). NDM-5 and NDM-1 are similar; the only difference they demonstrated is that two amino acids have been replaced (Val88Leu and Met154Leu), resulting in NDM-5 exhibiting a high level of resistance to carbapenems and expanded-spectrum cephalosporins, and thus posing a severe threat to public health (Hornsey et al., 2011). Since then, NDM-5 has spread globally, such as China (Zhang L.P. et al., 2016), the United States of America (Rojas et al., 2017), Australia (Wailan et al., 2015), Egypt (Soliman et al., 2016), and Italy (Giufre et al., 2018).

Worryingly, in China, NDM-5 has been detected in various species of *Enterobacterales* across various cities (Zhang F. et al., 2016; Li et al., 2017; Mao et al., 2018; Sun et al., 2018). In the current study, we identified a clinical *M. morganii* isolate producing NDM-5 and performed phylogenetic analysis. Further, we investigated the drug resistance profile and plasmid characteristic analysis to depict the potential transmission mechanisms of *bla*_NDM–5_.

## Materials and Methods

### Strain Screening

Since January 2016, we collected various clinical samples from patients based at the First Affiliated Hospital of Zhejiang University in Hangzhou (FAHZU). The samples were spread on the surface of MacConkey agar (OXOID, Hampshire, United Kingdom) plates that contained 2 mg/L meropenem (Meilunbio, Dalian, China) for 18–24 h at 37°C for the preliminary screening of carbapenem-resistant *Enterobacterales* (CRE) isolates (CLSI, 2018). The CarbaNP test and modified carbapenem inactivation method (mCIM) with EDTA-modified carbapenem inactivation method (eCIM) were used to detect carbapenemase activity (CLSI, 2018). Identification of species was performed using both matrix-assisted laser desorption/ionization time-of-flight mass spectrometry (MALDI-TOF/MS) (Bruker Daltonik GmbH, Bremen, Germany) and 16S rRNA gene sequencing. Carbapenemase genes (*bla*_KPC_, *bla*_NDM_, *bla*_OXA–48_, *bla*_V IM_, and *bla*_IMP_) were identified using PCR and DNA sequencing as described previously (Zheng et al., 2015). Finally, *M. morganii* L241 isolate was detected and its details were described in the results.

### Antimicrobial Susceptibility Testing

*Morganella morganii* L241 isolate was tested for resistance, using the agar dilution method, against 17 antibiotics, which were piperacillin/tazobactam, cefotaxime, ceftazidime, cefepime, cefpirome, aztreonam, ertapenem, imipenem, meropenem, amikacin, tetracycline, fosfomycin, gentamicin, chloramphenicol, ciprofloxacin, levofloxacin, and trimethoprim/sulfamethoxazole. Results were interpreted following the guidelines of the CLSI document M100-S28 (2018) (CLSI, 2018). *E.coli* ATCC 25922 was used as a control.

### Location of *bla*_NDM–5_ Gene and Transferability of Plasmid Carrying *bla*_NDM–5_

The number and size of plasmid of *M. morganii* L241 were determined with the S1 nuclease pulsed field gel electrophoresis (S1-PFGE) method, as described previously (Zheng et al., 2015). Southern blotting and hybridization using DIG-labeled *bla*_NDM_-specific probe were performed to estimate the location of *bla*_NDM_ gene, while the transferability of NDM-carrying plasmid from the isolate was determined through the use of conjugation experiments, with rifampicin-resistant *E. coli* C600 as the recipient strain. Further to this, transconjugants were selected on Mueller-Hinton agar (OXOID, Hampshire, United Kingdom) plates that contained both 200 mg/L rifampicin (Meilunbio, Dalian, China) and 2 mg/L meropenem. Finally, a combination of MALDI-TOF/MS identification, *bla*_NDM_ gene detection and antimicrobial susceptibility testing of the transconjugants were performed in order to confirm whether the plasmid was successfully transferred to the recipient.

### Whole Genome Sequencing and *in silico* Analyses

Genomic DNA was extracted using the OMEGA Bacterial DNA kit (Omega Bio-tek, Norcross, United) and was then sequenced on both the llumina HiSeq 4000-PE150 (Illumina, San Diego, CA, United States) and the PacBio RS II platforms (Pacific Biosciences, California, United States). We created a complete genome sequence for *M. morganii* L241 using Unicycler (Wick et al., 2017) by combining our llumina sequencing reads with PacBio sequencing reads. By using Unicycler (Wick et al., 2017), raw llumina reads were assembled using SPAdes, semi-global alignment was then performed by aligning PacBio reads to the assembly data, the llumina sequenceing reads were finally used to polish the genome assembly with Bowtie2 and Pilon. Additionally, online tools^1^ were used to identify acquired antimicrobial resistance genes and replicon type of plasmid. This genome was annotated by the RAST server (Aziz et al., 2008), while the IS Finder database^2^ was used to identify transposon and IS elements. The circular image of multiple plasmids comparisons was generated by the BLAST Ring Image Generator (BRIG) (Alikhan et al., 2011). Finally, the comparison figures of the genetic context of *bla*_NDM–5_ on multiple plasmids were performed with a Python application Easyfig (Zheng et al., 2017).

### Comparative Genomic Analysis

Genome sequences for 41 strains of *M. morganii* were downloaded from Pathogen Detection^3^. These genomes, plus the *M. morganii* L241 genomic sequence, were then analyzed using Snippy^4^, a process in which raw reads were mapped against the reference *M. morganii* genome (no.ALJX00000000) (Chen et al., 2012). A phylogenetic tree based on concatenated, qualified single nucleotide polymorphisms (SNPs) was then performed using Harvest (Treangen et al., 2014). Characteristics of all the *M. morganii* strains included in this study are summarized in Supplementary Table S1.

### Accession Numbers

The genome sequences of both *M. morganii* L241 chromosome and plasmid pNDM5-L241 were deposited in the GenBank with the accession numbers CP033056 and CP033057.

## Results

### Isolation and Identification of NDM-5-Producing *M. morganii* L241 Strain

A male patient of 53 years old was admitted to surgical ward of FAHZU in January 2016 and initially diagnosed as recurrence of liver cancer. The patient received hepatectomy 2 days after hospitalization and developed acute diarrhea on the third day after surgery. Since then, diarrhea has always existed. On the sixth day after surgery, a rod shaped Gram-negative bacterium, designated as L241, was recovered from the selective medium, which was found to be positive for the CarbaNP test, and mCIM with eCIM assay. Then it was confirmed as *M. morganii* and found to harbor *bla*_NDM–5_ after PCR and sequencing_._

### Antimicrobial Susceptibility Testing

The minimum inhibitory concentration (MIC) values of antimicrobials for *M. morganii* L241 are shown in Table 1. *M. morganii* L241 exhibited resistance to almost all of the β-lactam antibiotics tested, including piperacillin/tazobactam (MIC > 128 mg/L), cefotaxime (MIC > 128 mg/L), ceftazidime (MIC > 128 mg/L), cefepime (MIC = 128 mg/L), cefpirome (MIC > 128 mg/L), ertapenem (MIC = 32 mg/L), imipenem (MIC > 32 mg/L), and meropenem (MIC > 32 mg/L), with the exception of aztreonam (MIC = 1 mg/L). In addition, the isolate also demonstrated resistance to fosfomycin (MIC = 256 mg/L) and chloramphenicol (MIC = 32 mg/L) but was shown to be susceptible to amikacin, tetracycline, gentamicin, ciprofloxacin, levofloxacin, and trimethoprim/sulfamethoxazole.

**Table 1:** MIC values of antimicrobials for *M. morganii* L241, recipient strain *E. coli* C600 and transconjugant L241-*E. coli* C600.

Antimicrobials	MIC values (mg/L)		
	*M. morganii* L241	L241-*E. coli* C600	*E. coli* C600
Piperacillin/tazobactam	>128	>128	4
Cefotaxime	>128	>128	<0.25
Ceftazidime	>128	>128	1
Cefepime	128	128	<0.25
Cefpirome	>128	>128	<0.25
Aztreonam	1	0.25	0.5
Ertapenem	32	32	<0.015
Imipenem	>32	>32	0.5
Meropenem	>32	16	<0.015
Amikacin	8	2	2
Tetracycline	2	0.5	0.5
Fosfomycin	256	0.5	1
Gentamicin	4	1	1
Chloramphenicol	32	2	2
Ciprofloxacin	0.06	0.125	0.5
Levofloxacin	0.25	0.5	0.5
Trimethoprim/sulfamethoxazole	<0.125	<0.125	<0.125

*MIC, minimum inhibitory concentration.*

### Genomics Features of *M. morganii* L241

The genomic features of the *M. morganii* L241 are shown in Table 2. It was found that the *M. morganii* L241 genome consists of a 3,850,444 bp circular chromosome with an average G+C content of 51.1% and one plasmid. Further, the chromosome contained 3,831 protein coding genes, 82 tRNAs, and 22 rRNAs. A screening for acquired resistance determinants found that chromosome possess the resistance gene *catA2* encoding phenicol resistance and *bla*_DHA–17_ encoding β-lactam resistance; while the plasmid encoding acquired resistance gene confers resistance to β-lactams (*bla*_NDM–5_). This finding is consistent with the drug resistant phenotypes.

**Table 2:** Genomic features of the *M. morganii* L241.

Feature	Chromosome	pNDM5-L241
Total number of bases (bp)	3,850,444	46,161
G+C content (%)	51.1%	46.6%
No. of protein-coding sequences	3831	64
No. of rRNA genes	22	0
No. of tRNA genes	82	0
plasmid replicon type	–	IncX3
Resistance genes	*catA2, bla*_DHA–17_	*bla*_NDM–5_
Accession numbers	CP033056	CP033057

### Comparative Genomic Analysis

While previous studies have reported the genomes of *M. Morganii* (Khatri et al., 2013; Nash and Young, 2015), there are few studies which have linked the genetic information of multiple strains of *M. morganii* to explore their evolutionary relationships and the internal structure of the genome. Therefore, we performed a comparative genomic analysis.

**FIGURE 1:**
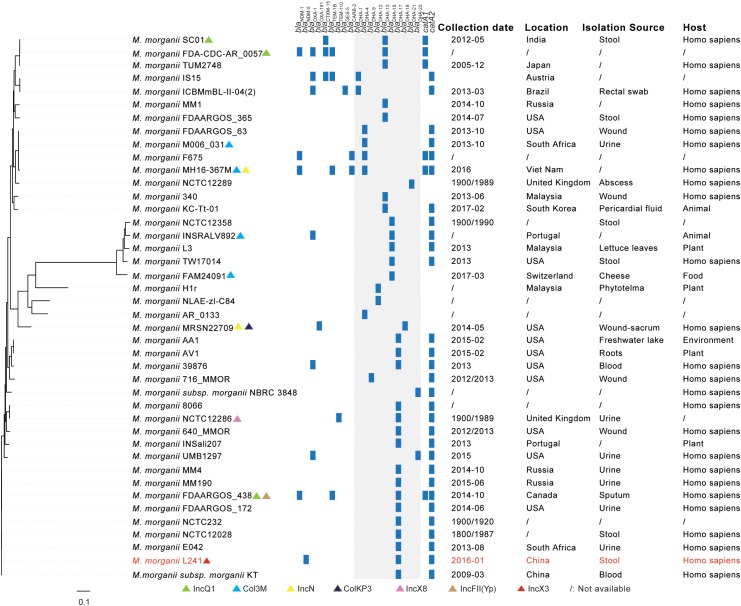
A comparative genome analysis of NDM-5-producing *Morganella morganii* L241 and other *M. morganii* isolates based on SNPs. The plasmid replication types, β-lactamases resistance genes, *catA* genes, collection dates, locations, isolation sources, and hosts of isolates are shown. The annotation denotes the presence of plasmid replication types, β-lactamases resistance genes and *catA* genes as determined by online tools (http://www.genomicepidemiology.org/). The *M. morganii* L241 is indicated by red. Regions with *bla*_DHA_ gene among isolates are shown by gray shading. The detail information of isolates included in this study is summarized in Supplementary Table S1.

As shown in Figure 1 and Supplementary Table S1, all of *M. morganii* isolates were found in various specimen types, including stool, rectal swab, wound, urine, blood, sputum, abscess, pericardial fluid, lettuce leaves, cheese, phytotelma, freshwater lake and roots from different hosts (homo sapiens, animal, plant, food, and environment). At the same time, they were detected across various countries, including India, Japan, Austria, Brazil, Russia, United States, South Africa, Viet Nam, United Kingdom, Malaysia, South Korea, Portugal, Switzerland, Canada, and China from 1800 to 2017, suggesting that *M. morganii* isolates are widely distributed. Resistance genes present on all *M. morganii* isolates showed that there was a common β-lactamases resistance gene *bla*_DHA._ In addition, no plasmid replication type was detected in most *M. morganii* isolates.

In addition to *M. morganii* NCTC12358, *M. morganii* INSRALV892, *M. morganii* L3, *M. morganii* TW17014, *M. morganii* FAM24091, *M. morganii* H1r, *M. morganii* NLAE-zl-C84, *M. morganii* AR_0133, and *M. morganii* MRSN22709, *M. morganii* L241 has close genetic relationships with other *M. morganii* isolates, among which *M. morganii* L241 is clustered with *M. morganii* MM4, *M. morganii* MM190, *M. morganii* FDAARGOS_438, *M. morganii* FDAARGOS_172, *M. morganii* NCTC232, *M. morganii* NCTC12028, *M. morganii* E042, and *M. morganii subsp. morganii* KT (Chen et al., 2012). These nine clustered strains were isolated from different specimen types and were detected at different times and in different countries. Further analysis of genomic information showed that all of these strains contained *bla*_DHA–17_ and *catA2* genes, but there was no common plasmid replicon type. It is noteworthy that *M. morganii* L241 and *M. morganii subsp. morganii* KT, which is the first genome sequence of *M. morganii*, are the most closely related isolates, differing by just 107 SNPs.

### Characterization of Plasmid Bearing *bla*_NDM–5_

**FIGURE 2:**
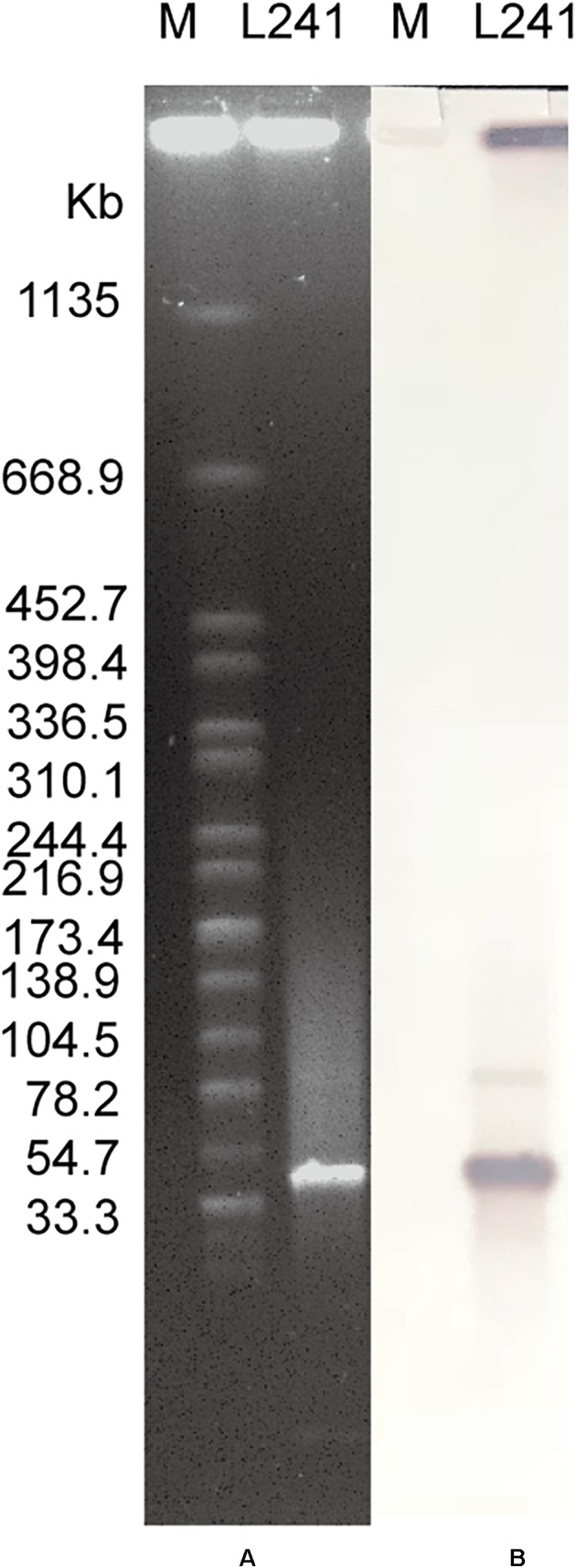
Plasmid profiles of *M. morganii* L241. **(A)** Plasmid size determination by S1-PFGE, with *Salmonella enterica* serotype Braenderup H9812 as the size marker. **(B)** Southern blotting hybridization with a *bla*_NDM_-specific probe.

The S1-PFGE result showed that only a ∼46-Kb plasmid was found in *M. morganii* L241 (Figure 2A). Subsequently, Southern blotting revealed that the *bla*_NDM–5_ gene was located on this plasmid (Figure 2B). PCR and sequencing analysis confirmed a transconjugant as *bla*_NDM–5–_encoding *E. coli* C600. This transconjugant exhibited resistance to almost all β-lactams aside from aztreonam, including piperacillin/tazobactam (MIC > 128 mg/L), cefotaxime (MIC > 128 mg/L), ceftazidime (MIC > 128 mg/L), cefepime (MIC = 128 mg/L), cefpirome (MIC > 128 mg/L), ertapenem (MIC = 32 mg/L), imipenem (MIC > 32 mg/L), and meropenem (MIC = 16 mg/L) (Table 1), with considerable increases in the MICs of carbapenems when compared with the recipient strain *E. coli* C600. These results indicate that the *bla*_NDM–5_-encoding plasmid of *M. morganii* L241, designated as pNDM5-L241, was successfully transferred into recipient *E. coli* C600 strain. In addition, results suggest that this was a self-transmissible plasmid. Owing to *M. morganii* L241 only possessing plasmid pNDM5-L241, the antimicrobial resistance phenotypes of the transconjugant were acquired from pNDM5-L241.

**FIGURE 3:**
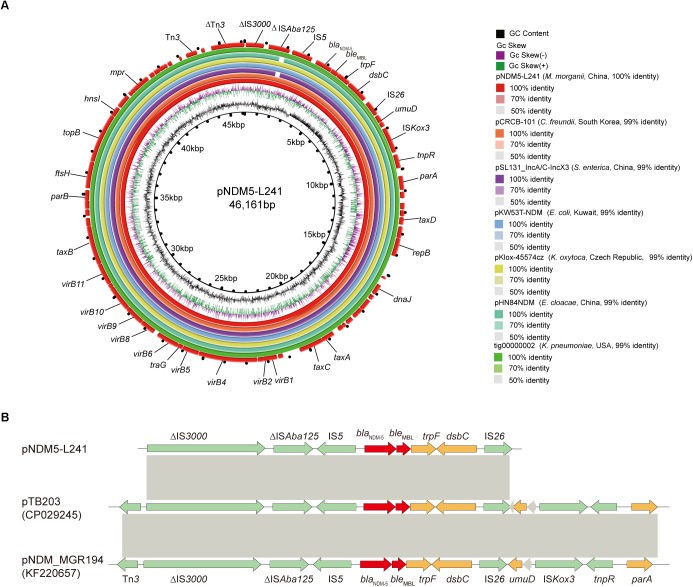
The genomic analysis of pNDM5-L241 plasmid. **(A)** Comparison of the pNDM5-L241 plasmid with the closely related plasmid based on BLASTn analyses (GenBank accession numbers from inside to outside are CP024820, MH105050, KX214669, MG833406, KY296103, and CP021759). **(B)** Genetic context of *bla*_NDM–5_ on pNDM5-L241, pTB203 (CP029245) and pNDM_MGR194 (KF220657). Open reading frames (ORFs) are represented by arrows and colored in accordance with their putative functions: red arrows indicate antimicrobial resistance genes, green arrows represent mobile genetic elements, while genes encoding hypothetical proteins and proteins for plasmid stability are colored as gray and brown, respectively. Regions with a high degree of homology between plasmids are shown by gray shading.

*In silico* analysis identified that plasmid pNDM5-L241 is an IncX3 type plasmid, with 46,161 bp in length, 64 predicted coding sequences and a GC content of 46.6%. This plasmid was home to several types of genes, such as antimicrobial resistance genes, mobile elements genes, putative genes, genes encoding replication proteins and genes encoding proteins for plasmid stability and plasmid transfer, respectively. A search of the nr/nt database found plasmid pNDM5-L241 exhibiting 99% nucleotide identity with the IncX3 *bla*_NDM–5_ encoding plasmid pTB203 (no. CP029245) and pNDM_MGR194 (no. KF220657). In all of these cases, *bla*_NDM–5_ was the only antimicrobial resistance gene. Importantly, a conserved structure sequence (ΔIS*3000*-ΔIS*Aba125*-IS*5*-*bla*_NDM–5_-*ble*-*trpF*-*dsbC*-IS*26*) was found in the upstream and downstream of the *bla*_NDM–5_ (Figure 3B).

Following this, we compared pNDM5-L241 with the *bla*_NDM–5_-encoding plasmid pKlox-45574cz (*Klebsiella oxytoca*, Czech Republic, no. MG833406), *bla*_NDM–7_-encoding plasmid pKW53T-NDM (*E. coli*, Kuwait, no. KX214669) (Pal et al., 2017), *bla*_NDM–5_-encoding plasmid pCRCB-101_1 (*Citrobacter freundii*, South Korea, no.CP024820), *bla*_NDM–1_-encoding plasmid pSL131_IncA/C-IncX3 (*Salmonella enterica subsp. enterica serovar Lomita*, China, no.MH105050), *bla*_NDM–1_-encoding plasmid pHN84NDM (*Enterobacter cloacae*, China, no.KY296103), and *bla*_NDM–7_-encoding plasmid tig00000002 (*Klebsiella pneumoniae*, United States, no.CP021759), which demonstrated a sequence similarity of 99% with coverages of 100, 100, 99, 99, and 100%, respectively (Figure 3A). As shown in Figure 3A, we found that the backbone sequences of the seven plasmids were almost identical.

## Discussion

*Morganella morganii* has been recognized as an increasingly important pathogen because of the increased frequency and a high mortality rate of its infections. In addition, according to a recent report, acquired resistance is increasingly observed in *M. morganii* (Liu et al., 2016). For example, *M. morganii* has shown resistance to β-lactams, aminoglycosides, phenicols, macrolides, tetracycline, trimethoprim, and fluoroquinolones (Liu et al., 2016). As a result of the intrinsic and acquired resistance of *M. morganii*, it poses a serious clinical threat which has limited treatment options. Nevertheless, there has not been too much attention on *M. morganii* so far.

The rapid development of gene sequencing technology has enabled us to have a deeper understanding of bacteria. As we know, the comparative genomic analysis based on SNPs combined with the plasmid replication type, antibiotic resistance gene content, time of isolation, geographical region, isolation source, and host is a valuable tool to conduct genomic epidemiological analyses. Therefore, in this study, the complete genome sequence and comparative genomic analysis were performed. Our analysis showed that *M. morganii* L241 is clustered with *M. morganii* MM4, *M. morganii* MM190, *M. morganii* FDAARGOS_438, *M. morganii* FDAARGOS_172, *M. morganii* NCTC232, *M. morganii* NCTC12028, *M. morganii* E042, and *M. morganii subsp. morganii* KT (Chen et al., 2012). The clustering phenomena and relatively small number of SNPs seen in these *M. morganii* isolates from different geographic locations over such a long time frame suggest that these isolates might be highly clonal. Further analysis showed that the clustering of *M. morganii* L241 with other *M. morganii* isolates was not determined by IncX3 type plasmid. Moreover, results showed that *M. morganii* L241 and *M. morganii subsp. morganii* KT are the most closely related isolates, prompting that *M. morganii* L241 may have evolved from *M. morganii subsp. morganii* KT. The pathogenicity-related factors of *M. morganii subsp. morganii* KT were identified, such as fimbrial adhesins, T3SS, TCS, iron acquisition system, IgA protease, and insecticidal and apoptotic toxins (Chen et al., 2012), implying *M. morganii* L241 has the similar toxicity characteristics.

To date, NDM-5 has been found in *Proteus mirabilis* (Zhang F. et al., 2016), *K. pneumonia* (Cho et al., 2015), *E. coli* (Soliman et al., 2016), *Enterobacter aerogenes* (Ahmad et al., 2018), and *Salmonella enterica* serovar Typhimuriumstrain (Li et al., 2017). As far as we are aware, the current study is the first report that has identified NDM-5 in *M. morganii*. This is a worrying development as it demonstrates the further spread of *bla*_NDM–5_ among different species of *Enterobacterales*.

In this work, we observed that a conserved structure sequence (ΔIS*3000*-ΔIS*Aba125*-IS*5*-*bla*_NDM–5_-*ble*-*trpF*-*dsbC*-IS*26*) was found in the upstream and downstream of the *bla*_NDM–5_ in IncX3 type plasmid. Interestingly, the conserved structure sequence is consistent with the upstream and downstream of the *bla*_NDM–5_ in IncFII type plasmid (Li et al., 2017). Recently, research has proposed that IS26 element may contribute to the vertically transfer of *bla*_NDM–5_ gene among plasmids and chromosomes (Li et al., 2017).

Horizontal gene transfer also contributed to the widespread dissemination of *bla*_NDM–5_ in *Enterobacterales*. Previously, the *bla*_NDM–5_ gene had been identified on various plasmid types, such as IncFII and IncX3 (Feng et al., 2018; Giufre et al., 2018). However, it has thus far been predominantly associated with IncX3 plasmids (Li et al., 2018). IncX3 plasmids carrying *bla*_NDM–5_ have spread widely among *Enterobacterales* worldwide (Li et al., 2018). These findings suggest that the production of *bla*_NDM–5–_ harboring *M. morganii* may be the result of the transmission of *bla*_NDM–5_*_-_* harboring IncX3. Additionally, it has recently been shown that *bla*_NDM–5–_ harboring IncX3-type plasmid isolated from raw milk and fecal samples from cows has spread among cow farms, suggesting that *bla*_NDM–5–_ harboring IncX3-type plasmid also can be transmitted from animals to humans through the food chain (He et al., 2017). This is an important finding. However, even more importantly, our BLASTn analysis showed that IncX3 plasmids harboring various *bla*_NDM_ genes, including *bla*_NDM–1_ (Lü et al., 2018; Zhu et al., 2018), *bla*_NDM–4_ (Espedido et al., 2015; Sugawara et al., 2017), *bla*_NDM–5_ (Krishnaraju et al., 2015; Zhu et al., 2016; Li et al., 2018; Xie et al., 2018)_,_ *bla*_NDM–6,_ *bla*_NDM–7_ (Pal et al., 2017; Sugawara et al., 2017), *bla*_NDM–13_ (Lv et al., 2016), *bla*_NDM–17_ (Liu et al., 2017), *bla*_NDM–19_ (Liu et al., 2019), *bla*_NDM–20_ (Liu Z. et al., 2018), and *bla*_NDM–21_ (Liu L. et al., 2018), which have been found in 15 species among *Enterobacterales* [*K. pneumoniae* (Espedido et al., 2015; Krishnaraju et al., 2015)*, K. oxytoca* (Paskova et al., 2018; Yoon et al., 2018), *Klebsiella michiganensis, Klebsiella aerogenes, E. coli* (Zhu et al., 2016; Pal et al., 2017; Sugawara et al., 2017; Li et al., 2018; Xie et al., 2018)*, C. freundii* (Zhu et al., 2018)*, S. enteric, Enterobacter hormaechei, Enterobacter cloacae* (Lü et al., 2018)*, Enterobacter asburiae* (Paskova et al., 2018)*, Enterobacter xiangfangensis* (Paskova et al., 2018)*, Cronobacter sakazakii, Raoultella planticola, Raoultella ornithinolytica* (Paskova et al., 2018), and *Kluyvera intermedia* (Paskova et al., 2018)] from 13 different countries around the world thus far. These countries are China (Zhu et al., 2016; Li et al., 2018; Liu Z. et al., 2018; Xie et al., 2018), the Czech Republic (Paskova et al., 2018), Kuwait (Pal et al., 2017), Korea (Yoon et al., 2018), Oman (Pal et al., 2017), United States, Sweden, Myanmar (Sugawara et al., 2017), Vietnam, India (Krishnaraju et al., 2015), Arabian Peninsula (Pal et al., 2017), Canada, and Australia (Espedido et al., 2015). These troubling results suggest that IncX3 type plasmids have attributed to the dissemination of the NDM variant among different species around the world. Of note, the IncX3 plasmid usually also bears other β-lactamase genes (*bla*_SHV_, *bla*_ampC_, *bla*_TEM_, *bla*_OXA_, and *bla*_KPC_) and encodes resistance genes which are responsible for other antibiotics, such as quinolones (*qnr*), sulphonamides (*sul1*), and tetracyclines (*tet*) (Dobiasova and Dolejska, 2016; Cerdeira et al., 2017; Bitar et al., 2018). Taken together, the transmission of this plasmid may lead to a severe threat to public health. It is crucial that we take urgent and effective measures to control the dissemination of the IncX3 type plasmids.

## Conclusion

In summary, we first identified a *bla*_NDM–5_-positive *M. morganii* and reported its complete genome sequence. The *bla*_NDM–5_ gene was located on a self-transmissible IncX3 plasmid which spread among species of *Enterobacterales* worldwide. This study highlights the wide spread of *bla*_NDM_-encoding IncX3 plasmids, including their transmissionin to uncommon *Enterobacterales* strains including *M. morganii*. Therefore, the IncX3 plasmids must be closely monitored, and attention must be paid to uncommon *Enterobacterales* strains. Further research is necessary to prevent and control the spread of *bla*_NDM_-encoding IncX3 plasmids.

## Data Availability

The datasets generated for this study can be found in NCBI, CP033056 and CP033057.

## Ethics Statement

Written informed consent was obtained from the participants of this study.

## Author Contributions

XG and BZ conceived and designed the experiments. YR, HX, TL, YC, NL, and HH performed the experiments. LG and XY analyzed the data. BZ and YR wrote the manuscript.

## Conflict of Interest Statement

The authors declare that the research was conducted in the absence of any commercial or financial relationships that could be construed as a potential conflict of interest.

## Footnotes

1 http://www.genomicepidemiology.org/

2 https://www-is.biotoul.fr/

3 https://www.ncbi.nlm.nih.gov/pathogens/

4 https://github.com/tseemann/snippy
