# Supplementary material for: Detection and Genomic Characterization of a Morganella morganii Isolate From China That Produces NDM-5
Source: Front Microbiol. 2019 May 28;10:1156. doi: 10.3389/fmicb.2019.01156 (PMC6546717; doi:10.3389/fmicb.2019.01156)
Supplement: Supplementary file 1 [file Table_1.DOCX]

**Table S1.** Characteristics of all *M. morganii* strains included in comparative genomic analysis.

| Organism | Strain | BioSample | BioProject | Assembly | AMR genotypes | Plasmid replication type | Collectiondate | Location | Isolation Source | Host |
| --- | --- | --- | --- | --- | --- | --- | --- | --- | --- | --- |
| *M. morganii* | SC01 | SAMN01822804 | PRJNA175933 | GCA_000307755.2 | *aadA5, aph(3'')-Ib, aph(6)-Id, armA, bla*_CTX-M-15_*, bla*_DHA-13_*, catA1, dfrA17, mph(E), msr(E), sul1, sul2* | IncQ1 | 2012-05 | India | Stool | Homo sapiens |
| *M. morganii* | FDA-CDC-AR_0057 | SAMN04014898 | PRJNA316321 | GCA_002968775.1 | *aac(3)-IIa, aac(6')-Ib-cr, aadA2, aadA5, aph(3')-Ia, aph(3'')-Ib, aph(6)-Id, bla*_CTX-M-15_*, bla*_DHA-13_*, bla*_NDM-1_*, bla*_OXA-1_*, bla*_TEM-1B_*, catA1, catB3, dfrA12, dfrA17, mph(A), sul1, sul2, tet(B)* | IncQ1 | / | / | / | / |
| *M. morganii* | TUM2748 | SAMD00115702 | PRJDB6851 | GCA_003176395.1 | *aph(3')-Ia, ant(2'')-Ia, bla*_DHA-13_*, catA1, sul1, tet(B)* | - | 2005-12 | Japan | / | Homo sapiens |
| *M. morganii* | IS15 | SAMEA3138874 | PRJEB4780 | GCA_000530115.1 | *aac(3)-IIa, aac(3)-IId, aac(6')-Ib-cr, bla*_CTX-M-15_*, bla*_DHA-1_*, bla*_OXA-1_*, bla*_TEM-1B_*, catA2,* *catB3, erm(42), dfrA15, sul1, sul2* | - | / | Austria | / | / |
| *M. morganii* | ICBMmBL-II-04(2) | SAMN06562966 | PRJNA361484 | GCA_002029935.1 | *aac(6')-Ib3, aac(6')-Ib-cr, aadA2, bla*_DHA-1_*, bla*_GES-5_*, bla*_OXA-1_*, catA2, catB3, dfrA15, mph(E), msr(E), sul1, tet(B)* | - | 2013-03 | Brazil | Rectal swab | Homo sapiens |
| *M. morganii* | MM 1 | SAMN09771590 | PRJNA484881 | GCA_003390295.1 | *bla*_DHA-13_ | - | 2014-10 | Russia | / | Homo sapiens |
| *M. morganii* | FDAARGOS_365 | SAMN07312409 | PRJNA231221 | GCA_002386305.1 | *bla*_DHA-13_ | - | 2014-07 | USA | Stool | Homo sapiens |
| *M. morganii* | FDAARGOS_63 | SAMN02934512 | PRJNA231221 | GCA_000783955.2 | *bla*_DHA-4_*, tet(B), catA2* | - | 2013-10 | USA | Wound | Homo sapiens |
| *M. morganii* | M006_031 | SAMN06106880 | PRJNA355910 | GCA_002417235.1 | *aadA7, bla*_DHA-4_*, catA2, qnrD1, sul1, tet(B)* | Col3M | 2013-10 | South Africa | Urine | Homo sapiens |
| *M. morganii* | F675 | SAMEA2602632 | PRJEB6425 | GCA_000752335.1 | *aadA2, aph(3')-VI, bla*_CARB-2_*, bla*_DHA-4_*, bla*_NDM-1_*, catA2, catA1, dfrA19, ere(A), ere(B), mph(A), tet(B)* | - | / | / | / | / |
| *M. morganii* | MH16-367M | SAMD00112925 | PRJDB6655 | GCA_003114875.1 | *aadA2, ant(2'')-Ia, aph(3')-Ia, rmtB, bla*_CARB-2_*, bla*_DHA-4_*, bla*_NDM-1_*, bla*_TEM-1B_*, catA2, catA1, catB3, dfrA19, qepA1, qnrD1, sul1, tet(A), tet(B)* | Col3M, IncN | 2016 | Viet Nam | / | Homo sapiens |
| *M. morganii* | NCTC12289 | SAMEA104200655 | PRJEB6403 | GCA_900453195.1 | *bla*_DHA-21_ | - | 1900/1989 | United Kingdom | Abscess | Homo sapiens |
| *M. morganii* | 340 | SAMN02983093 | PRJNA258045 | GCA_000747035.1 | *bla*_DHA-13_ | - | 2013-06 | Malaysia | Wound | Homo sapiens |
| *M. morganii* | KC-Tt-01 | SAMN08335225 | PRJNA429323 | GCA_002891475.1 | *bla*_DHA-13_*, catA2* | - | 2017-02 | South Korea | Pericardial fluid | Animal |
| *M. morganii* | NCTC12358 | SAMEA104338361 | PRJEB6403 | GCA_900453145.1 | *bla*_DHA-16_*, catA2, tet(D)* | - | 1900/1990 | / | Stool | / |
| *M. morganii* | INSRALV892 | SAMN03944814 | PRJNA291471 | GCA_001263435.1 | *aac(6')-Ib-cr, aadA1, aph(6)-Id, aph(3'')-Ib, aph(3')-Ia, bla*_DHA-16_*, bla*_OXA-1_*, qnrD1, catA2, catB2, catB3, dfrA1, floR, sul2, tet(D), tet(Y)* | Col3M | / | Portugal | / | Animal |
| *M. morganii* | L3 | SAMN03145039 | PRJNA265008 | GCA_001006565.1 | *aadA1, bla*_DHA-16_*, catA2, dfrA1, tet(D)* | - | 2013 | Malaysia | Lettuce leaves | Plant |
| *M. morganii* | TW17014 | SAMN03813741 | PRJNA288592 | GCA_001274995.1 | *bla*_DHA-16_*, catA2, tet(D)* | - | 2013 | USA | Stool | Homo sapiens |
| *M. morganii* | FAM24091 | SAMN08142803 | PRJNA421410 | GCA_003034205.1 | *bla*_DHA-16_*, tet(D)* | Col3M | 2017-03 | Switzerland | Cheese | Food |
| *M. morganii* | H1r | SAMN02729859 | PRJNA226209 | GCA_000633515.1 | *bla*_DHA-12_*, tet(D)* | - | / | Malaysia | Phytotelma | Plant |
| *M. morganii* | NLAE-zl-C84 | SAMN05216301 | PRJEB18384 | GCA_900142745.1 | *bla*_DHA-12_*, tet(D)* | - | / | / | / | / |
| *M. morganii* | AR_0133 | SAMN04014974 | PRJNA292902 | GCA_003071325.1 | *aadA1, bla*_DHA-4_*, dfrA1, tet(D)* | - | / | / | / | / |
| *M. morganii* | MRSN22709 | SAMN03075590 | PRJNA261723 | GCA_000770295.1 | *bla*_DHA-18_*, bla*_OXA-181_*, dfrA14, qnrS1* | ColKP3, IncN | 2014-05 | USA | Wound-sacrum | Homo sapiens |
| *M. morganii* | AA1 | SAMN06446007 | PRJNA376738 | GCA_002077675.1 | *bla*_DHA-17_*, catA2* | - | 2015-02 | USA | Freshwater lake | Environment |
| *M. morganii* | AV1 | SAMN06446189 | PRJNA376739 | GCA_002077705.1 | *bla*_DHA-17_*, catA2* | - | 2015-02 | USA | Roots | Plant |
| *M. morganii* | 39876 | SAMN06218048 | PRJNA339843 | GCA_002180575.1 | *aadA24, bla*_DHA-17_*, bla*_OXA-1_*, catA2, dfrA1, sul1* | - | 2013 | USA | Blood | Homo sapiens |
| *M. morganii* | 716_MMOR | SAMN03197921 | PRJNA267549 | GCA_001066005.1 | *aac(3)-IId, aadA5, bla*_DHA-9_*, catA2, dfrA17, mph(A), sul1, tet(B)* | - | 2012/2013 | USA | Wound | Homo sapiens |
| *M. morganii* | NBRC 3848 | SAMD00046901 | PRJDB430 | GCA_001598895.1 | *bla*_DHA-22_*, catA2* | - | / | / | / | Homo sapiens |
| *M. morganii* | 8066 | SAMN03375434 | PRJNA252708 | GCA_000966695.1 | *bla*_DHA-17_*, catA2* | - | / | / | / | Homo sapiens |
| *M. morganii* | NCTC12286 | SAMEA104200656 | PRJEB6403 | GCA_900453165.1 | *bla*_DHA-17_*, bla*_TEM-110_*, catA2, tet(B)* | IncX8 | 1900/1989 | United Kingdom | Urine | / |
| *M. morganii* | 640_MMOR | SAMN03197841 | PRJNA267549 | GCA_001066745.1 | *bla*_DHA-17_*, catA2* | - | 2012/2013 | USA | Wound | Homo sapiens |
| *M. morganii* | INSali207 | SAMN04497995 | PRJNA311932 | GCA_001653675.1 | *aadA1, ant(2'')-Ia, bla*_DHA-17_*, mph(A), catA2, cmlA1, sul1, tet(B)* | - | 2013 | Portugal | / | Plant |
| *M. morganii* | UMB1297 | SAMN08193707 | PRJNA316969 | GCA_002847885.1 | *aadA1, aadA5, aac(6')-Ib-cr, bla*_DHA-22_*, bla*_OXA-1_*, catA2, catB3, dfrA17, sul1, tet(B)* | - | 2015 | USA | Urine | Homo sapiens |
| *M. morganii* | MM 4 | SAMN09695391 | PRJNA482068 | GCA_003340585.1 | *bla*_DHA-17_*, catA2* | - | 2014-10 | Russia | Urine | Homo sapiens |
| *M. morganii* | MM 190 | SAMN09509789 | PRJNA478302 | GCA_003287815.1 | *bla*_DHA-17_*, catA2* | - | 2015-06 | Russia | Urine | Homo sapiens |
| *M. morganii* | FDAARGOS_438 | SAMN07312482 | PRJNA231221 | GCA_002588265.1 | *rmtC, aac(3)-IId, aadA5, aph(3'')-Ib, aph(6)-Id, bla*_DHA-17_*, bla*_NDM-1_*, bla*_TEM-1B_*, mph(A), catA1, catA2, dfrA17, sul1, sul2, tet(B)* | IncFII(Yp), IncQ1 | 2014-10 | Canada | Sputum | Homo sapiens |
| *M. morganii* | FDAARGOS_172 | SAMN03996316 | PRJNA231221 | GCA_001558895.2 | *aadA1, bla*_DHA-17_*, catA2, dfrA1, sul2, tet(B)* | - | 2014-06 | USA | Urine | Homo sapiens |
| *M. morganii* | NCTC232 | SAMEA4552889 | PRJEB6403 | GCA_900453135.1 | *bla*_DHA-17_*, catA2* | - | 1900/1920 | / | / | / |
| *M. morganii* | NCTC12028 | SAMEA2709025 | PRJEB6403 | GCA_900478755.1 | *bla*_DHA-17_*, catA2* | - | 1800/1987 | / | Stool | Homo sapiens |
| *M. morganii* | E042 | SAMN06106837 | PRJNA355910 | GCA_002416605.1 | *bla*_DHA-17_*, catA2* | - | 2013-08 | South Africa | Urine | Homo sapiens |
| *M. morganii* | KT | SAMN02603113 | PRJNA78681 | GCA_000286435.2 | *bla*_DHA-17_*, catA2, tet(B)* | - | 2009-03 | China | Blood | Homo sapiens |

/: Not available.

-: Not detect.
